# Pilot study: Improving attention bias modification of alcohol cues through concealed gaze-contingent feedback in alcohol dependence

**DOI:** 10.1016/j.abrep.2019.100231

**Published:** 2019-11-06

**Authors:** Timo L. Kvamme, Mads U. Pedersen, Morten Overgaard, Kristine Rømer Thomsen, Valerie Voon

**Affiliations:** a Centre for Alcohol and Drug Research, School of Business and Social Sciences, Aarhus University, Aarhus, Denmark; b Department of Psychiatry, University of Cambridge, Cambridge, United Kingdom; c Cognitive Neuroscience Research Unit, CFIN/MindLab, Aarhus University, Aarhus, Denmark; d Behavioral and Clinical Neurosciences Institute, University of Cambridge, Cambridge, United Kingdom; e NIHR Biomedical Research Council University of Cambridge, United Kingdom

**Keywords:** Attentional bias modification, Eye-tracking, Alcohol, Gaze-contingent feedback, Craving

## Abstract

In an attempt to improve attention bias modification (ABM), we tested whether an attentional training protocol which featured monetary operant conditioning of eye-gaze to avoid alcohol stimuli in alcohol-dependent patients could reduce attention, craving and relapse to alcohol. We employed a pilot randomized control trial (RCT) with 21 detoxified alcohol dependent patients (48.9 ± 10 years of age, 9 male) from an inpatient and outpatient treatment centre. The novel concealed operant conditioning paradigm provided monetary reinforcements or punishments respective to eye-gaze patterns towards neutral or towards alcohol stimuli along with an 80% probability of a to-be-detected probe appearing following neutral stimuli (ET-ABM group). Patients in the control-group received random monetary feedback and a 50/50 ABM contingency. We compared AB on trained and untrained stimuli and addiction severity measures of obsessive thoughts and desires to alcohol following training. We further assessed addiction severity and relapse outcome at a 3-month follow-up. Results indicate that this attentional retraining only worked for the trained stimuli and did not generalize to untrained stimuli or to addiction severity measures or relapse outcome. Potential explanations for lack of generalization include the low sample size and imbalances on important prognostic variables between the active-group and control-group. We discuss progress and challenges for further research on cognitive training using gaze-contingent feedback.

## Highlights

• Prior research shows that attentional biases to alcohol stimuli can be retrained.
• This experimental study added concealed operant conditioning of eye-gaze patterns.
• Results indicate effects only for the trained stimuli and did not generalize to untrained stimuli.

## 1 Introduction

Incentive-motivational models of addiction propose that variations in substance abuse maps onto biased cognitive processing of disorder-related stimuli (Franken, 2003, Robinson and Berridge, 2001). During acquisition of addictive alcohol behaviours, discriminative stimuli such as the sight and smell of alcohol become repeatedly paired with the rewarding outcome of alcohol consumption. Consequently, the now conditioned stimuli or “cues” are said to have acquired attention grabbing properties because they have become associated with an expectation of the rewarding alcohol consumption (Field and Cox, 2008, Gilpin and Koob, 2008, Robinson and Berridge, 2001, Wiers et al., 2016).

One way to measure this “attentional bias” (AB) to alcohol-related cues is through the visual-probe task (VPT) where key-press latencies are generally faster to a to-be-detected target probe if it appears following an alcohol-related stimulus rather than a neutral stimulus (Field & Eastwood, 2005). It has been suggested that AB has a bi-directional causal relationship with alcohol craving and that alcohol dependent patients may benefit from cognitive therapies aimed at reducing AB (Field & Cox, 2008). This is the goal of attention bias modification (ABM) which has been shown to reduce AB by presenting the target probe more often following the neutral stimulus (Field et al., 2013, Mathews and MacLeod, 2002, Schoenmakers et al., 2010). Despite some putative therapeutic effects, results of reaction-time-based ABM protocols for improving treatment outcome in alcohol dependence remains decidedly mixed (Christiansen et al., 2015, Wiers et al., 2018). Moreover, an outstanding problem regarding ABM is that of generalizability to new (unhabituated) alcohol-related stimuli, e.g. stimuli that was not used during training (Field et al., 2007).

Strategies for enhancing the effect of ABM are in all likelihood more fruitful if guided by a thorough understanding of its mechanism of action. Based on their seminal study on ABM, Mathews and MacLeod (2002) proposed that inducing processing biases in the VPT may rely on a learned production rule: if two valenced stimuli appear, select the neutral. One could argue that ABM may work by biasing attention towards neutral stimuli since attending to the neutral stimuli results in faster reactions to the subsequent probe. Faster reactions to the probe may be intrinsically rewarding, because it speeds up the task with which the individual is engaged. In operant conditioning terms, the neutral and salient stimuli are discriminant stimuli, where attendance towards neutral as a response is reinforced through the subsequent reward, thereby increasing the probability of that response (Skinner, 1945). The pairing of neutral stimuli with an intrinsically rewarding feature should, in principle, be akin to pairing the neutral stimuli with an extrinsic reward, such as monetary stimuli. Following this logic, we reasoned that adding monetary rewards to the ABM paradigm after overt attention is measured on neutral stimuli could enhance the effects of ABM. Eye-tracking based measures could more accurately shape the behavior as eye-tracking provides a continuous measurement of overt attention, with gaze movements typically sampled 60 times per second or more. Eye-tracking measures might also provide better therapeutic targets compared with reaction-time (RT) based ABM protocols because key-press behavior occurs down-stream from a dynamically evolving attentional process (Armstrong and Olatunji, 2009, Christiansen et al., 2015). Information from gaze-movements have been successfully deployed in other domains to enhance visual-search strategies and thus may provide a new method of bias change (Carroll et al., 2013, MacLeod et al., 2009, Sadasivan, 2005).

As preliminary proof-of-concept, the present pilot-study tested a novel ABM paradigm which added concealed operant conditioning of eye-gaze patterns on stimuli in the visual-probe paradigm. Detoxified alcohol dependent patients were randomly assigned to either ABM, which featured additional monetary feedback contingent on their eye-gaze avoidance of alcohol-related stimuli (ET-ABM group), or a visual-probe ABM-control condition featuring monetary rewards unrelated to their eye-gaze (control-group). We hypothesized that patients allocated to the active group would show decreased AB following the intervention, which would generalize to untrained (e.g. unhabituated) stimuli. We further hypothesized decreases of cue-induced craving scores at group level and tested for lasting effects of the intervention on addiction symptomology (obsessive thoughts and desires for alcohol) in addition to abstinence over a 3-month period.

## 2 Method

### 2.1 Participants

Twenty-one participants (M = 48.9 ± 9.7 age, 9 male) were recruited from one inpatient and one outpatient treatment centre in Denmark as part of another study (Kvamme, Rømer Thomsen, Pedersen, Overgaard, & Voon, 2019). All patients were screened by expert clinicians at their respective treatment centre and fulfilled ICD-10 diagnostic criteria for alcohol dependence as part of the inclusion criteria. The mean Alcohol Use Disorder Identification Test (AUDIT) (Saunders, Aasland, Babor, de la Fuente, & Grant, 1993) scores, which tracks problematic alcohol use the year before initiation of treatment, were 28.9 ± 6.7, with a score of 20 being a useful cut-off point for considering further diagnosis for alcohol dependency (see Table 1 for details). One month prior to treatment, patients in the study reported having been intoxicated on average 5.3 ± 1.8 days per week with an average of 25.7 ± 15.9 units (UK) of alcohol per day.

**Table 1:** Characteristics of participants allocated to the active ET-ABM and control groups at the pre-test.

	Active ET-ABM Group (n = 11), mean (SD)	Control Group (n = 10), mean (SD)
**AB 500 ms**	5.29 (20.5)	−11.1 (31.9)
**AB 1200 ms**	−3.4 (9.3)	−7.6 (19.6)
**Age**	50.3 (8.6)	47.4 (10.6)
**Gender (F/M)**	7/4	5/5
**AUDIT**	29.3 (8.3)	28.4 (4.2)
**BDI**	14.9 (11.3)	16.3 (6.4)
**AUQ**	150.4 (98.5)	127.4 (52.8)
**OCDS**	8.6 (6.1)	9.2 (4.6)
**DAQ**	**2.1 (0.8)**	**3.4 (1.2)**
**Craving**	2.5 (3.15)	2.2 (2.6)

Note. N = 21, Significant difference between groups in **boldface.***AB* Attention Bias, *OCDS* Obsessive Compulsive Drinking Scale, *DAQ* Desire for Alcohol Questionnaire, *AUQ* Alcohol Use Questionnaire, *AUDIT* Alcohol Use Disorder Identification Test, *BDI* Becks Depression Inventory.

All patients provided informed consent to participate in the study, and all procedures of the study were approved by the local ethics committee in accordance with the 1964 Helsinki declaration. Patients were paid 50 £ to take part in the two testing and five training sessions at their respective treatment centre and 15 £ for the follow-up phone interview. Patients were not told that the intention of the experiment was to change their attitudes towards alcohol-related stimuli. Patients reported having been detoxified for at least 1 week before testing (M = 10 ± 13 weeks). Before each session, patients were breathalysed using a Lifeloc FC10 (Lifeloc Technologies, Colorado, US). All patients had an undetectable breath alcohol level at each session. Patients were tested over a 3-to-4-week period on days they were able to partake in the study.

### 2.2 Questionnaires

**Fig. 1:**
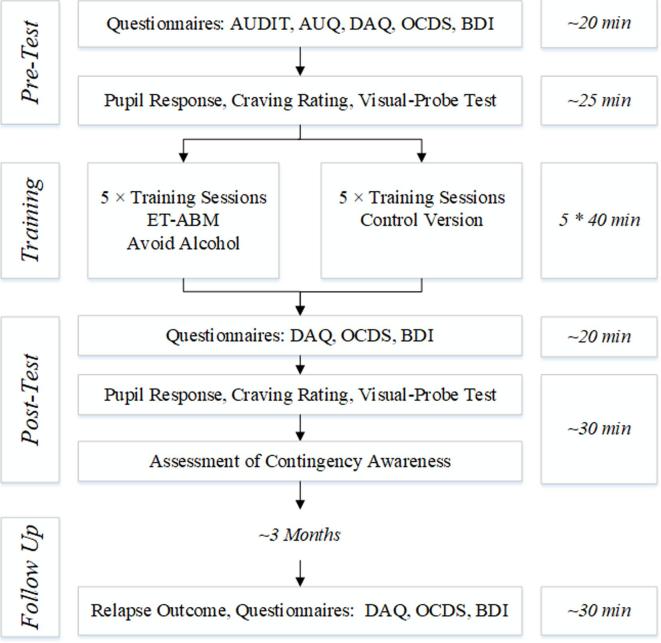
Schematic of experimental tests and questionnaires. AUDIT Alcohol Use Disorder Identification Test, AUQ Alcohol Use Questionnaire, DAQ Desire for Alcohol Questionnaire, OCDS Obsessive Compulsive Drinking Scale, BDI Becks Depression Inventory; ET-ABM; Eye-Tracking Attention Bias Modification.

Patients completed questionnaires accompanied by an experimenter. All questionnaires, except for questionnaires on prior alcohol use and prior problematic alcohol use, were completed twice: before and after the attentional training on days with no training (see Fig. 1 for an overview of the flow of experimental tests and questionnaires).

*AUDIT.* Prior problematic use of alcohol was measured using the Alcohol Use Disorder Identification Test (AUDIT) (Saunders et al., 1993), which consists of a 10-item questionnaire developed as a screening instrument for hazardous and harmful alcohol consumption. Patients were asked to provide answers relating to their drinking patterns one year before enrolment in treatment.

*AUQ.* Prior alcohol use was measured using the Alcohol Use Questionnaire, which assesses quantity of alcohol consumption within the last month (Mehrabian & Russell, 1978). Patients were asked to provide answers relating to their drinking patterns one month before enrolment at the treatment centre.

*DAQ*. Craving for alcohol was assessed with the Desires for Alcohol Questionnaire (DAQ) (Love, James, & Willner, 1998), which is a 14-item questionnaire consisting of Likert scales with a range of 1 (low desire for alcohol) to 7 (high desire for alcohol).

*OCDS.* Obsessive thoughts about alcohol was measured using the Obsessive Compulsive Drinking Scale (OCDS) (Anton, Moak, & Latham, 1995). The OCDS is a 14-item questionnaire consisting of Likert scales with a range of 1–5. Low scores indicate that a person rarely has occurring thoughts about alcohol and a high degree of control over thoughts relating to alcohol while high scores relate to frequent thoughts about alcohol that have an intrusive and uncontrollable characteristic.

*BDI.* Depressive tendencies were assessed with Becks Depression Inventory (BDI) (Beck, Steer, & Carbin, 1988).

### 2.3 Cue-reactivity task & craving scores personalization

Following questionnaires, participants performed a cue-reactivity task where they rated a series of alcohol images. These images served to provide individualized control of the subset of stimuli used for training and new stimuli used for the post-test. Participants were seated in front of a 17-in. laptop monitor (1920 × 1080 resolution, 60 Hz) with a Tobii x2-60 Eye tracker providing a bilateral sample (each 16.6 ms) of eye positions and pupillary diameter. The task commenced upon a 6-point calibration procedure using Tobii Studio software at 60 cm viewing distance. The stimulation interface was custom programmed in Python using PsychoPy (Version 1.84.2) (Peirce, 2007). During the cue-reactivity task, participants were presented with 48 alcohol images obtained from the internet representing the alcohol choices typically available in Denmark. Alcohol images were interspersed by 48 neutral images obtained through a standardized picture set. The neutral images were matched approximately for color, size, form, complexity and brightness to the alcohol images (Blechert, Meule, Busch, & Ohla, 2014). Following a fixation cross, participants were presented with either an alcohol or neutral image on the centre of the screen for 2000 ms during which participants were asked to fixate on the image. Participants were queried for their rating of the alcohol images (craving rating). Participants were asked to rate their temptation to drink the specific alcohol shown under an imagined scenario where they were outside the clinical setting and would allow themselves to drink.

The 48 alcohol images and their matched 48 neutral images were divided into two subsets categories of “trained images” used during the training sessions and “untrained images” presented only once during rating and at the subsequent post-test of AB. To control for craving rating and pupillary reactions between the subsets we performed an automated selection of the two subsets that sought to minimize the difference in craving rating and mean pupillary diameter recorded during the entire 2000 ms presentation. Specifically, we performed a quantile split based on each participant’s rating of the images into a (1): very low (2): low (3): high and (4): very high rating. Within each quantile we generated combinations (n = 1000) of the allocation of images to either subset and choose the combination where the image subset categories were least explained (lowest Z score) by the independent variables of craving ratings and mean differential pupillary diameter in separate logistic regression models for each combination.

### 2.4 Alcohol visual-probe task

**Fig. 2:**
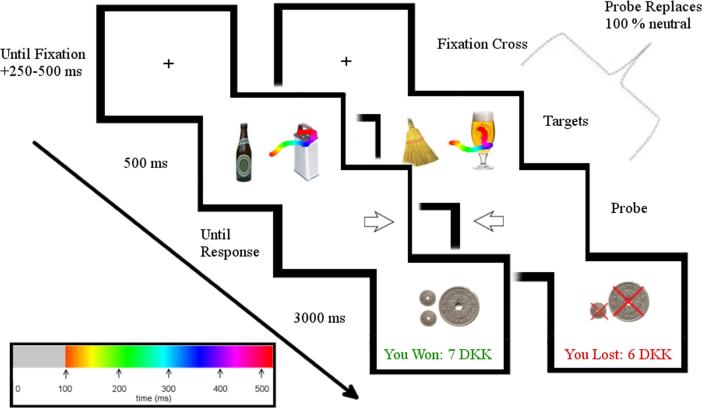
Attention Bias Modification with eye-tracking based feedback trials. Two examples of trials for participants allocated to the eye-tracking based feedback (ET-ABM) group. Left; the participant’s gaze was on the neutral image and thus positively reinforced. Right; the participant’s gaze was on the alcohol cue and thus negatively punished. Gaze movements are depicted for analytical clarity and was not shown to participants in the experiment. The color of the gaze movements is shown in a spectrum of color signifying the time course corresponding to the bottom left legend. ms: milliseconds, DKK: local currency of Danish Crowns. (For interpretation of the references to color in this figure legend, the reader is referred to the web version of this article.)

Participants performed a visual-probe task (VPT) modified for alcohol (Field and Eastwood, 2005, Field et al., 2007) which featured either “trained” or “untrained” images at the respective pre-test and post-test. Each trial proceeded as follows (see Fig. 2): Participants were asked to gaze at a central fixation cross, displayed for a randomized inter-trial-interval lasting between 750 and 1250 ms. The ending of the fixation phase was further contingent on eye-gaze measurement being within 1.5° of the fixation cross. An instruction to fixate on the cross was displayed if participants did not fixate on the cross within 2 s. The cross was replaced by a pair of neutral and alcohol images for a cue duration of either 500 ms or 1200 ms. After picture offset, a probe (arrow pointing left or right) appeared behind one of the images for 250 ms. Participants were asked to indicate by keypress (“z” or “m”) the position of the probe as fast as possible, and reaction times (RTs) were recorded. After detailed introduction and 10 practice trials participants performed the experimental trials in which pictures were randomized such that each of the 24 pictures appeared in each side of the display, cue duration and the position preceded by the probe. This yielded a total of 192 trials (24 × 2 × 2 × 2).

### 2.5 Allocation

Participants were allocated to the active and control group to match for prognostic factors using a custom sequential covariate adaptive minimization technique. We first calculated their pre-test attentional bias to alcohol stimuli by subtracting their response latencies to alcohol images from those to neutral images. For each two participants enrolled in the study we constructed two separate hypothetical allocations (i.e.: active (ET-ABM) and control or reversed). From this we calculated the explained variance of the two separate logistic regression models with group as the dependent variable and age, gender along with pre-test scores of BDI, AUDIT and AB as covariates. At each enrolment following the first participant, the program would automatically choose the allocation where the total variance in the aforementioned covariates explained the least variance in group allocation. With this procedure, we attempted to minimize the difference between groups in prognostic baseline factors to ward off assignment bias due to chance imbalance while also blinding assignment to the experimenter (Scott, McPherson, Ramsay, & Campbell, 2002).

### 2.6 Eye tracking-based feedback and attention bias modification

The intervention consisted of five sessions where the active group performed the ABM procedure accompanied by eye tracking-based feedback (ET-ABM) while the control group performed a control task. Compared with the test version of the VPT, the attentional training program differed in cue duration, the location of the probes for the active group and featured an additional monetary feedback phase. The cue duration of neutral and alcohol images was presented consistently at 500 ms (trained cue duration). The probe replaced the neutral images on 80% of the trials for participants allocated to the ET-ABM group and 50% for participants in the control group. Immediately after cue offset, the participants’ eye fixations on - and eye movements in relation to - the images during the 500 ms viewing period were calculated in near real-time while participants were responding to the probe. The later 400 ms SOA was used, as the first 100 ms have been shown to be contingent on anticipatory reactions (Fischer & Weber, 1993). The conditioning schedule was designed so that each gaze position counted proportionally towards a larger or smaller positive reinforcer or negative punishment based on the saliency of the stimuli. For a sample of gaze positions captured within the image boundaries the program added 4 positive or negative “points” depending on saliency of neutral and alcohol cues respectively. Eye movements towards or away from the alcohol image counted between 0 and 4 positive or negative points depending on the velocity, with a maximum of 40 degrees per second yielding 4 points (Mogg, Millar, & Bradley, 2000). The points were converted into monetary stimuli with a 10 DKK reward or loss signifying an aggregate point score above or below +70/−70. Points between 70 to −70 led to 7 DKK reward to −7 DKK losses at each 10-point/1 DKK interval. Erroneous answers to the probe position resulted in a −5 DKK loss in both groups which was unaffected by gaze position (1.7% of all trials). The monetary feedback stimuli were presented for 3 s following responses to probe position.

Every 40th trial participants were offered to take a break, where their aggregate monetary earnings were shown. Participants were instructed that they would receive a proportion of the money they earned through the task, at the final test. All participants received the same amount. The contingencies of the feedback for a given participant were unbeknownst to both participant and experimenter. The participant was told that “you will do the same as the previous trials (referring to 10 initial practice trials similar to the pre-test), except that you will now receive feedback based on (1): how fast and, (2); how accurately you responded to the probe and (3): how stable your fixation was on the initial fixation cross. Participants in the study were told that the aim of study was to measure reaction times to alcohol stimuli over a series of days and that the feedback was there to motivate them. The monetary feedback stimuli provided to participants in the control group was unrelated to their eye movements, as they received the monetary feedback “yoked” from the first participant enrolled in the study. The first participant’s allocation to the active group was unblinded to the experimenter. In summary, the active group condition aimed to unconsciously train the participants’ eye-gaze away from alcohol images using probe contingencies and eye-tracking-based feedback. The comparison group was used to control for the effect of habituation to the stimuli and the inclusion of monetary feedback in the active group. Participants performed the trials in randomized order in blocks of 10 with each of the 24 images appearing counterbalanced in each side (10 × 24 × 2) resulting in 480 trials for each five training sessions.

### 2.7 Contingency awareness questionnaire and confidence token game

Following the last VPT task, participants completed a contingency awareness questionnaire (CAQ) which aimed to assess their level of cognitive awareness of the contingencies on the probe location (adopted from Grafton, Mackintosh, Vujic, & MacLeod, 2013), along with questions assessing their awareness of the relationship between their eye-gaze and monetary feedback. It was emphasized to the participant that the experimenter could only provide guidance as the experimenter was unaware of the contingencies. First, participants were asked to guess the probability of how often the probe replaced the neutral image on a scale from 100%, to −0% in 20% intervals on the five sessions from pre- to post-test. Secondly, the participants were asked in open ended questions what they thought the money they received or had taken away from them during the five training sessions was related to and which if any strategy they employed to earn more money during the task. To further examine the degree to which participants were aware of the relationship between their eye movements and monetary feedback we developed a Confidence Token Game (CTG). Participants were given six red rectangular tokens (representing their confidence) and were faced with a 6 × 6 grid where the columns of the grid had six behaviours supposedly related to the monetary reward in the task. The behaviours were; (1): fixation on the initial fixation cross, (2); correctly answering probe position, (3): quickly answering probe position, (4): looking more at neutral images than alcohol images, (5): looking more at alcohol images than neutral images, and (6): the reward was random. Participants were required to place all six tokens within the columns of one or several of the behaviours they thought were related to increased reward and were allowed to relocate the tokens. Participants were told that more than one column (behavior) could be correct and that some could be incorrect. For all questions above and the CTG, participants also rated their confidence in their answer on a scale from 0 to 100.

### 2.8 Relapse rates

Three months following the post-test assessment, patients were contacted by phone for information about their current relapse status. Similar to our previously published study detailing the relationship between pupillary reactions to alcohol stimuli and relapse (Kvamme et al., 2019), we choose to code relapse outcome using three categories of increasing severity of values (No Relapse = 0, Minor Relapse = 1 Major Relapse = 2). Major relapse was defined as drinking behavior persisting for more than a week as opposed to minor relapse referring to any drinking (see Reyes-Huerta, Vacio, Pedroza, Salazar, & Martínez, 2018 for a review on relapse definitions).

## 3 Results

*Pre-treatment characteristics.* Table 1 shows a summary of data of pre-intervention variables for participants allocated to the active ET-ABM and control group. Group comparisons on demographic variables and pre-intervention factors were calculated as t-statistics for quantitative measures and chi-square statistics for gender. The control group had a significantly higher DAQ score than the ET-ABM group [t (16) = −2.70, P = 0.01] at the pre-test. The groups did not differ on any other pre-intervention variable (p > 0.6). RTs from the alcohol VPT were excluded if they were above 2000 ms or below 200 ms and trials with erroneous indication of probe position (3.1% of data). Attentional bias scores were calculated by subtracting the median RT from trials where probes replaced alcohol cues from those where probes replaced neutral images. There was no pre-test difference between active and control groups on AB for either cue durations of 500 ms (P = 0.22) or 1200 ms (P = 0.54). All 21 patients included in the study completed the intervention, however one participant in the control group left the treatment centre for undisclosed reasons and did not complete post-test measures nor follow-up.

*Attention Bias Training Effects.* We tested the overall effect of ET-ABM compared to the control group using analyses of covariance (ANCOVAs) on post-test scores with pre-test scores entered as a covariate to increase statistical power (Van Breukelen, 2006). As groups differed on DAQ scores at pre-test, we performed ANCOVA’s with the mean of the DAQ scores as a covariate in subsequent analyses. We performed analyses separately for the two image sets (‘trained’ images that were used in attentional training, and ‘untrained’ images, not used in attentional training) and the two cue durations of 500 ms of 1200 ms.

**Fig. 3:**
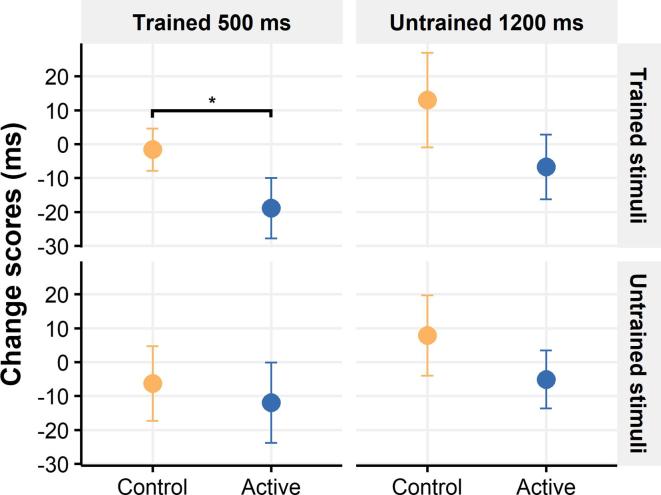
Change scores (in ms) from the visual-probe task at pre-test to post-test (values below zero indicate decreases in AB across time). Data shown for the control group “orange color” and active ET-ABM group “blue color” separately for each condition; trained stimuli (used during attentional training) and “untrained’ stimuli (not used during attentional training) and the two cue durations of 500 ms (trained cue duration) and 1200 ms (untrained cue duration). Values are mean ± SEM. * p < 0.05. (For interpretation of the references to color in this figure legend, the reader is referred to the web version of this article.)

ANCOVA on the post-test scores for trained image set in the trained cue duration of 500 ms revealed a main effect of group [F (1,11) = 4.918, P = 0.048], however the interaction of DAQ, pre-test AB scores and Group was also significant [F (1,11) = 29.45, p < 0.001] indicating that a higher initial DAQ score in the control group confounded the comparison of attentional training effect between groups. The main effect of group was confirmed, using within-subject *t* tests on the trained image set and cue duration revealing that AB scores decreased significantly in the active ET-ABM group (M = 18.9 ms (95% CI 2.69 to 38.8)) [t (10) = 2.25, p _one-tailed_ = 0.024] and not the control group (M = 1.2 ms (95% CI −10.2 to 15.6)) [t (8) = 0.21, p _one-tailed_ = 0.41] (see Fig. 3).

The ANCOVA on the post-test scores in the trained cue duration showed that the groups did not differ for untrained images [F (1,11) = 1.3, P = 0.27], nor was any main effect or interaction significant. There were no significant main effects or interactions on ANCOVAs on the untrained cue duration of 1200 ms for either trained images or untrained images.

*Craving.* There was no pre-test difference between groups on craving ratings as indicated by a Mann–Whitney analysis (P = 0.8). The ANCOVA on post-test scores showed no group effects on craving ratings [F (1,11) = 0.33, P = 0.57]. However, an effect of initial DAQ score [F (1,11) = 13.67, P = 0.003] and an interaction between DAQ and pre-test craving scores [F (1,11) = 5.83, P = 0.03] were found. To test whether changes in AB related to changes in craving scores, we calculated AB change scores as the difference between post-test AB and the pre-test AB scores for each cue duration (Allison, 2016). Change scores for all analyses were calculated as post-test minus the pre-test, such that a negative score represents decrease in AB scores over time.

A correlation analysis revealed that AB and craving rating did not correlate at pre-test or at the post-test [r = 0.17, P = 0.46] and [r = −0.25, P = 0.28] respectively. However, the changes in AB related to a change in craving rating [r = 0.53, P = 0.015], as AB tended to decrease, so too did craving scores. We performed a linear regression model to test whether DAQ scores influenced the relationship between AB change scores and change in craving ratings. Analyses revealed that higher initial DAQ scores related to a decrease in craving ratings [t (16) = −3.67, P = 0.002] and an interaction between DAQ scores and AB change scores [t (16) = 2.97, P = 0.009], confirming an unfortunate confound of DAQ scores on craving score changes.

*Obsessive Compulsive Drinking Scale.* ANCOVAs on OCDS questionnaire scores showed no main effect of group at the post-test [F (1,17) = 0.001, P = 0.97] nor at the 3-month follow-up [F (1,17) = 1.89, P = 0.19] respectively.

*Contingency Awareness.* Whether participants had awareness of the experimental contingencies was investigated with data from the CAQ. The degree of awareness of the contingencies for probe replacement was tested for differences between groups by means of t-tests, revealing no difference between groups [t (19) = −0.42, P = 0.68]. A correlation analysis in the active group revealed no significant relationship [r = 0.36, P = 0.25] between contingency awareness and overall changes in AB, but was stronger within the trained cue duration [r = 0.43, P = 0.15], albeit still not significant. The participant-provided placements of tokens in the CTG were evaluated. Only two participants placed tokens indicating that the feedback-reward was related to eye movements away from alcohol, one from the control group (placing four tokens, 66% confidence), and the other from the active group (placing two tokens, 33% certainty). In the open-ended questions, only the person in the control group answered that the feedback was related to less eye movements to alcohol, whereas the person in the ET-ABM group stated that the feedback was meant to distract.

### 3.1 Exploratory analyses

*Power calculations.* For the individual comparisons of cue durations and image sets, we calculated the effect size using Cohen’s effect size *d* (pooled variance) as the difference between the two groups in AB change scores across time (Lakens, 2013). From this, we derived the size *n* required in each group necessary to have 0.80 power to detect an effect below 0.05 alpha with a directed hypothesis of decreased AB in the active group. For the trained images and cue duration this was [*d =* 0.69, *n* = 26] and untrained cue duration [*d =* 0.54, *n* = 43] whereas for the untrained images and trained cue duration [*d =* 0.15, *n* = 512] and untrained cue [*d =* 0.40, *n* = 74].

*Relapse outcome.* Of the 20 patients who completed the intervention and post-test measures we reached 18 patients for a follow-up on relapse status (10 ET-ABM, 8 control). Three patients in the ET-ABM group (30%) and one control-group patient (12%) experienced a major relapse, while three ET-ABM patients (30%) and two control-group patients (25%) experienced a minor relapse. An ordinal regression model with the three relapse categories as outcome and group as independent variable showed that the ET-ABM group had a higher [z = 1.03, P = 0.3] degree of relapse, albeit non-significant.

*Pupillary Reactions Baseline.* To investigate possible factors explaining the trend towards higher degree of relapse in the active group compared to the control group, we conducted exploratory analyses on the pupillary reactions to the alcohol stimuli at the pre-test. Our prior study investigated the differential pupillary reactions to alcohol images versus neutral images (“pupillary bias”) and its relationship to relapse outcome (Kvamme et al., 2019). The previously published paper details a relationship between relapse and the differences in pupillary diameter measured in the 150-to-250 ms time window after viewing the brightness-controlled alcohol and neutral images. For our present study, we extracted the differential pupillary reactions within the specified time window and compared the two groups by means of a between-subjects *t* test. Pupillary reactions to alcohol images indeed did differ by group at baseline [t (16) = 2.48, P = 0.024], indicating that the active group had a higher pupillary bias to alcohol images at baseline. This baseline was not significantly related to AB change [r = 0.29, P = 0.23].

## 4 Discussion

The principle finding of this pilot study is that ABM coupled with eye-tracking based operant conditioning of gaze behavior reduces AB on the trained images within the trained target duration. No significant effects were found on other AB-components, nor on craving to the specific alcohol images, addiction symptomology following the intervention or at 3-month follow-up. Reductions in AB across time were related to decreases in craving scores to alcohol stimuli although this effect was not larger for the active ET-ABM group.

Given that the objective of the intervention was to test eye-tracking based operant conditioning as an adjunct to ABM, the preliminary results provide proof-of-concept and some encouragement for the use of an eye tracking-based attention retraining protocol. A major concern before the initiation of the study was whether participants in the active group saw through the masked intention of the feedback (concealment of contingencies). Participants’ awareness of contingencies was manipulated in another study, showing that attentional bias effects are present regardless of cognitive awareness of contingencies, although the generalization to more therapeutically meaningful measures is decreased when contingencies are known (Grafton et al., 2013). A major strength of our study was therefore the extended investigation into participants’ awareness of contingencies and the result that patients generally did not become aware of contingencies of the eye-tracking feedback. The retention rate of participants throughout the study was also an important concern. Only one participant allocated to the control group of all 21 patients was unable to partake in the post-test measures. Furthermore, only two participants were unreachable at follow-up. Thus, only few participants did not complete the full study.

A notable strength of the current study is the use of craving ratings and pupillometry as a control for trained and untrained picture sets for each participant, where prior studies assume these measures to be equivalent (Field et al., 2007, Schoenmakers et al., 2010). It has been shown that personalized stimuli can impact the attentional bias measure in the VPT (Christiansen, Mansfield, et al., 2015).

We sought with our allocation of patients to active and control group to ward off chance imbalance on important preclinical measures by employing a novel covariate adaptive minimization technique. Despite our attempt to ensure that covariates of age, gender, BDI, AUDIT and AB scores were matched between groups we erroneously assumed that matching for AUDIT and AB would result in matching scores on OCDS and DAQ. Well-balanced groups are often achieved in larger clinical trials, however with smaller trials, such as the present, minimizing the differences between prognostic factors is crucial for a clear interpretation of the change in primary and secondary measures (Scott et al., 2002). The more two clinical groups differ on an important characteristic at baseline, the more room there is for one of them to regress towards the population mean, which can make it difficult to disentangle the effect of an intervention (Morton & Torgerson, 2003). In our case, a high baseline DAQ score in the control group could indicate a more severe group, which consequently had more potential room for regressing towards a “healthier” mean. This proved deleterious in our analysis, as the DAQ baseline scores interacted with group and pre-test AB scores when comparing the effect on post-test AB scores. Furthermore, there was an interaction between DAQ baseline and pre-test craving scores when estimating the group level effects on craving at the post-test. It is thus hard to distinguish if the difference in the experimental manipulations between the groups was inconsequential, or if the regression to the mean on DAQ impacted an improvement in the control group. If the latter, the regression to the mean of DAQ scores could have masked our ability to spot the hypothesized bigger improvement in the active group, such as a more generalized effect on AB (i.e. on untrained stimuli or untrained cue duration). Furthermore, we found no evidence for an intended effect on relapse rates within the active group, and our exploratory analyses confirmed that the groups were different on another important parameter related to relapse, namely pupillary responses. Our recent study (Kvamme et al., 2019) suggests pupillary reactions to alcohol stimuli is an important prognostic variable for relapse. Further studies on ABM should aim to match groups to a greater extent than achieved in our study. Based on the findings of our pilot study, we stress the need to match for DAQ scores when comparing effects on AB and craving scores, as well as pupillary reactions when comparing effects on alcohol relapse.

Our study is based on previously established links between AB and craving for alcohol (Field and Cox, 2008, Wiers et al., 2016). Contrary to this, we found that AB and craving as measured in our cue reactivity task did not correlate at the pre-test or at the post-test, although this may relate to our study being notably underpowered. Interestingly, we found that changes in AB across time related to changes in craving ratings. This specific measure of craving was associated with levels of alcohol use in a young non-clinical population (Kvamme et al., 2018). This is important for further CBM research as it lends support to the rationale that interventions that robustly modify AB to alcohol-related stimuli could be beneficial for alcohol-dependent patients (Karno, 2018, Schoenmakers et al., 2010).

Looking ahead, our power analyses revealed that significant effects on the trained images and trained durations (500 ms) as well as within the untrained (1200 ms) duration are achievable with a sample size of around 150 participants. Although caution should be taken when estimating sample sizes from pilot studies (Kraemer, Mintz, Noda, Tinklenberg, & Yesavage, 2006), our analyses point to the untrained images within the brief 500 ms duration as being a particularly small effect, thus requiring more than 1000 participants to achieve significance. In a large-scale study, Rinck, Wiers, Becker, and Lindenmeyer (2018) performed a comparison between an ABM protocol and an active control group on its effect on AB, approach bias and relapse at one-year follow-up. In the study by Rinck et al. (2018), a successful bias effect was achieved as AB only increased in the control group and not in the active ABM group. This was measured only on trained images. Importantly, relapse rates were higher in the control group compared with the active ABM group. Surprisingly however, the success rate in achieving abstinence was not predicted by the change in bias score, arguing against the necessity of AB change for effects on relapse risk.

Elucidating the underlying neurocognitive mechanism involved in ABM is critical for moving the field forward. Technical improvements to the ABM paradigm as we have investigated here should be encouraged in order to target a proposed mechanism of action. Specifically, unlike merely enhancing the reward of speeded reactions, feedback of detailed gaze-behavior could be able to selectively reward fixations versus saccades or provide differential shaping of eye-gaze in different time courses of stimulus presentation This is of particular relevance to the visual processing of alcohol stimuli, as the initial orientation (as compared to the later processing stages) relate to addiction status, and is more challenging to modify through traditional RT-based ABM (Noël et al., 2006, Schoenmakers et al., 2010). Thus, if successfully implemented, eye-tracking based feedback could greatly expand the toolset used to investigate and treat cognitive processing biases.

To conclude, this is one of the first studies coupling concealed eye-tracking based operant conditioning of gaze behavior with an ABM task, targeting attentional biases in recovering alcohol dependent patients. We provide proof-of-concept that this type of feedback can be delivered without participants’ knowledge. Despite this, our results indicate that this attentional retraining only worked for the trained stimuli and there was no evidence of generalization to untrained stimuli, nor therapeutically relevant effects on addiction severity measures or relapse outcome.

## Author contributions

T.L.K, K.R.T, and V.V designed the study; T.L.K, K.R.T, M.U.P recruited the patients; T.L.K performed the data collection; T.L.K analysed data; T.L.K, K.R.T, M.O and V.V interpreted the data; T.L.K wrote the manuscript; all authors edited and approved the final version of the manuscript.

## Declaration of Competing Interest

The Authors have no Conflict of Interest to Declare.
